# Actualización y manejo clínico de los anticuerpos anti-ácido desoxirribonucleico

**DOI:** 10.1515/almed-2020-0067

**Published:** 2021-03-02

**Authors:** Concepción González Rodríguez, M. Belén Aparicio Hernández, Inmaculada Alarcón Torres

**Affiliations:** Unidad de Bioquímica Clínica, Hospital Universitario Virgen Macarena Sevilla, Sevilla, España; Servicio Bioquímica Clínica y Análisis Cínicos, Complejo Asistencial Universitario Salamanca, Salamanca, España; Servicio Análisis Clínicos, Hospital Universitario Gran Canaria-HUGCDN, Gran Canaria, España

**Keywords:** anti-dsDNA, marcador clínico, lupus eritematoso sistémico

## Abstract

Los anticuerpos contra el ácido desoxirribonucleico [DNA] en el laboratorio clínico, están íntimamente ligados al diagnóstico y monitorización del lupus eritematoso sistémico [LES]; no obstante, las características de los métodos analíticos y las propiedades de los propios anticuerpos son heterogéneas Revisar la definición y propiedades de los anticuerpos anti-DNA de doble cadena [anti-dsDNA], la adecuación de los métodos analíticos y los requerimientos clínicos para este biomarcador. A través de PubMed se investiga la bibliografía existente con los términos anti-dsDNA, editorial, review, guideline, meta-analysis y LES. La última búsqueda, anti-dsDNA y LES restringuida a los últimos dos años. Se amplía información a través de artículos relacionados y los publicados en organismos oficiales estatales relacionados con anti-dsDNA y LES. Se analizan los métodos del laboratorio clínico para el análisis de los anti-dsDNA y sus características. Se revisa la utilidad clínica de los anti-dsDNA en sus aspectos diagnóstico, de asociación clínica y seguimiento del LES. Existe una amplia variabilidad en los métodos analíticos y persisten déficits en la estandarización. Forman parte de los criterios actuales clasificatorios de LES y se utilizan como marcadores en el seguimiento de la enfermedad. La utilidad diagnóstica mejora cuando se determinan en pacientes con ANA positivos. En el seguimiento, es interesante la cuantificación, preferiblemente con el mismo método analítico (dado los déficits de estandarización).

## Introducción

La baja prevalencia y un espectro amplio de síntomas y signos dificultan el diagnóstico de las enfermedades reumáticas autoinmunes sistémicas. Por ello, se precisan biomarcadores, tales como, los anticuerpos dirigidos contra el *ácido desoxiribonucléico* [DNA] [1].

Los anticuerpos contra el DNA pueden reconocer todas las estructuras del DNA presentes en la cromatina, tanto en su estado de reposo como activo. Reconocen secuencias de DNA, DNA lineal de cadena sencilla, DNA de doble cadena [dsDNA]bien circular o bien helicoidalen sus diferentes formas: BdsDNA, la más habitual con la doble hélice girando a la derecha, Z dsDNA girando hacia la izquierda, dsDNA elongado con la hélice alargada y dsDNA superenrollado y girando sobre sí mismo [2]. Sin embargo, no se han demostrado asociaciones clínicas para las subpoblaciones de anti-dsDNA dirigidas contra las estructuras descritas de anti-dsDNA [2]. En esta revisión se considerarán incluidas en el término general de anti-dsDNA. Por otro lado, los anti-ADNdc pueden ser de clase IgA, IgG e Ig M; pero los segundos son los más relevantes clínicamente y los que habitualmente se determinan en la práctica clínica [3].

El espectro de métodos analíticospara la determinación de anti-dsDNA es muy amplio y con diferentes características; lo que dificulta su utilización y significado clínico. Por ello, el objetivo de este trabajo se centra en revisar la definición y propiedades de los anticuerpos anti-dsDNA, analizar las propiedades analíticas y clínicas de los métodos de medida y aportar herramientas que faciliten el empleode anti-dsDNA como biomarcador en la atención del paciente con LES.

## Materiales y métodos

Se realizan varias investigaciones bibliográficas avanzadas en PubMed con los términos anti-dsDNA y tipo de publicación; considerando editorial, review, guideline o meta-analysis. Igualmente, se realiza una búsqueda restringida a los últimos dos años con los términos anti-dsDNA y SLE. Se amplía información a través de la búsqueda de artículos relacionados y material publicado en organismos oficiales estatales relacionados con anti-dsDNA y LES.

## Resultados

Se estructura la información revisada en cinco epígrafes: definición de anticuerpos anti-DNA y anti-dsDNA, propiedades, determinación, utilidad clínica y orientaciones de uso.

### Definición

El DNA constituye el principal componente del material genético y es el responsable de almacenar la información genética. En las células procariotas y eucariotas se empaqueta en una estructura denominada nucleosoma; constituida por DNA e histonas [4].

Anticuerpos anti-DNA son aquellos que reconocen a las diferentes estructuras o componentes del DNA. Sin embargo, desde el punto de vista clínico y a pesar de sus limitaciones, el más relevante es el anti-dsDNA [5].

Los pacientes con LES generan anticuerpos contra dsDNA, bien aislado, unido a proteínas (ej. histonas) o integrado en estructuras más complejas como los nucleosomas [4].

Los anticuerpos contra los nucleosomas y las histonas poseen ciertas implicaciones clínicas. Mientras que los anti-histonas se asocian a lupus inducido por droga; los anti-nucleosomas, poseen un significado clínico similar a los anti-dsDNA, pudiendo detectarse en fases tempranas del LES, con una implantación clínica es muy inferior [6], [7].

### Propiedades

Cuando se inicia una respuesta inmune humoral la afinidad de los anticuerpos es baja; pero conforme progresa, la afinidad del anticuerpo hacia su ligando [epítopo] se incrementa. En moléculas polivalentes, como son las inmunoglobulinas, se va incrementando, igualmente, la fuerza global con la que la molécula de inmunoglobulina interacciona con la molécula antigénica, a través de sus diferentes ligandos [avidez] [8]. En los centros germinales, se produce maduración y selección de la respuesta inmunológica que se manifiesta en las formas peptídicas de la región variable de las inmunoglobulinas [9].

**Figura 1:**
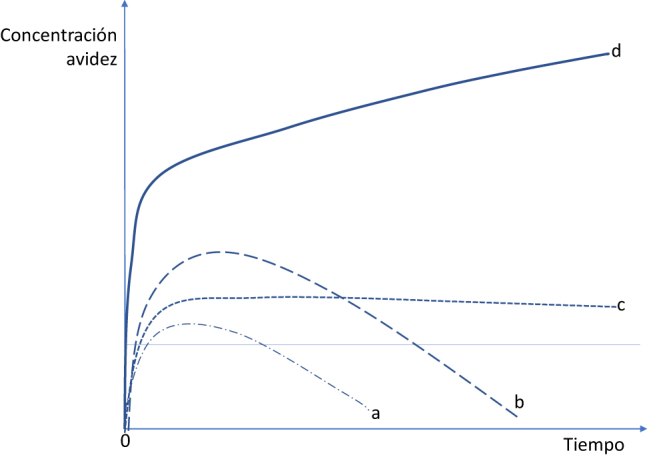
Perfiles teóricos de respuesta anti-dsDNA. (a) Uun estímulo ya sea una infección o autólogo origina una respuesta transitoria con concentraciones y avidez bajas de anti-dsDNA si es breve; (b) una respuesta transitoria pero más elevada cuando el estímulo es más fuerte; (c) unas concentraciones bajas pero persistentes de anti-dsDNA cuando el estímulo permanece; (d) una respuesta inmunológica con producción mantenida de anti-dsDNA a concentraciones y grado de avidez elevados en un contexto autoinmune adecuado. Modificada de Rekvig [10].

En un contexto inmunogénico normal, un estímulo, como puede ser una infección bacteriana o vírica, es capaz de inducir una respuesta inmunológica transitoria de defensa que reconozca al anti-dsDNA de la bacteria o del virus e incluso del propio huésped [Figura 1a]. Si el estímulo persiste, los anti-dsDNA persisten a concentraciones bajas [Figura 1b]. Si el estímulo es muy fuerte la respuesta transitoria es fuerte igualmente [Figura 1c]. Pero en un contexto autoinmune, la respuesta anti-dsDNA reconoce al anti-dsDNA propio y se mantiene y progresa; incrementándose las concentraciones, al igual que la afinidad y avidez. Esta última reacción es la más característica y específica del LES [Figura 1d] [10].

Junto al agente infeccioso, otros estímulos [apoptosis exacerbada, exposición a radiaciones ultravioleta, fármacos…] pueden generar anti-dsDNA en pacientes con LES, cáncer, u otras enfermedades autoinmunes [10], [11], [12]. Parece que los anti-dsDNA reconocenlas moléculas de DNA dañado con mayor afinidad y formando complejos inmunes más fuertes [13].

### Determinación

#### Métodos

Hasta hace pocos años los anticuerpos anti-dsDNA se han detectado mediante métodos inmunoenzimáticos de ELISA [*enzyme linked immunosorbent assay*] [14], inmunofluorescencia indirecta sobre el hemoflagelado *Crithidea Luciliae* [CLIFT] [15] y radioinmunoanálisis [16]; pero en las últimas décadas han surgido nuevos métodos como los inmunoanálisis enzimáticos fluorescentes [FEIA] [17], quimioluminiscentes [18] y los que analizan múltiples parámetros [anticuerpos] de forma simultánea [MPIA] [19], [20].

Los más utilizados en nuestro entorno son los métodos de FEIA y CLIFT, seguidos de EIA, CLIA y MPIA, de acuerdo a los informes metodológicos del programa de calidad UK-NEQAS que cuenta con más de 600 participantes [21]. Muy pocos laboratorios emplean inmunoblot o radioinmunoanálisis; ya que el primero no se recomienda [22] y el segundo ha sido sustituido por métodos que evitan el uso de radioisótopos [23].

La técnica de Farr, descrita en 1969 [24] ha sido considerada el método de referencia. Se caracteriza por ser cuantitativa y de especificidad elevada para el LES. La especificidad se atribuye al uso de altas concentraciones de sales [sulfato amónico] durante el paso de precipitación de los inmunocomplejos dsDNA/anti-dsDNA, lo que se cree, selecciona los anticuerpos de alta avidez [25]. Probablemente esta prueba posea la mejor relación sensibilidad/especificidad para el LES; pero usa radioisótopos y detecta anti-dsDNA de clase IgA, IgG e IgM sin poder diferenciar entre ellas. Recientemente, se ha descrito un método de Farr modificado que intercala un colorante fluorescente en la molécula dsDNA (Farr-FIA). Se correlaciona muy bien con el método de Farrradioisotópico y alcanza una sensibilidad y especificidad diagnósticas del 53% y 100%, respectivamente [26].

En la CLIFT el dsDNA nativo está altamente compactado en el quinetoplasto. Posee especificidad elevada con alto valor predictivo positivo; pero su sensibilidad es inferior a la de otros métodos [especialmente en LES temprano] [27].

En la década de los 80, comienza a utilizarse métodos totalmente automatizados, primero ELISA y después FEIA, MPIA y CLIA. Estos métodos poseen, en general, mayor sensibilidad y menor especificidad que CLIFT [detectando anti-dsDNA de afinidad y avidez inferiores]; aunque las características varían mucho entre los fabricantes. Algunos de ellos utilizan concentraciones elevadas de sales en el tampón de lavado, tanto en ELISA [28] como en CLIA [29]; habiéndose descrito, en el último, buenas relaciones sensibilidad/especificidad [18].

En los últimos años, se ha descrito un método de detección fluorimétrica que permite la cuantificación de anti-dsDNA libre e integrado en complejos inmunes circulantes (DNA endógeno-antidsDNA); incorporándolos últimos, a la detección y cuantificación habitual [30].

#### Características

Las características de los métodos comerciales más habituales aparecen reflejadas en la Tabla 1; habiéndose referenciado cada método al equipo más utilizado por los laboratorios que participan en el programa de calidad UKNEQAS: Orgentec [EIA], Phadia 250 [FEIA], INOVA Quanta Flash [CLIA] y LuminextechnologyBioRadBioplex2200 [MPIA] [21].

**Tabla 1:** Características de diferentes métodos analíticos para anti-dsDNA.

Método	Antígeno	Fase sólida	Conjugado	Tiempo análisis	Detección	Calibración	Rango analítico	Puntos corte
CLIA	dsDNA sintético	Bola para magnética	IgG	30 min	Cuantitativa	Curva	9.8–666.9 UI/mL	35–45 UI/mL equívoco >45 IU/mL positivo
MPIA	dsDNA sintético	Bola magnética coloreada	IgG	45 min	Cuantitativa	Curva	1–300 UI/mL	5–9 UI/mL dudoso ≥10 UI/mL positivo
FEIA	dsDNA sintético	Micro pocillo	IgG	120 min	Cuantitativa	Curva	0,5–379 UI/mL	10–15 UI/mL dudoso >15 UI/mL positivo
EIA	dsDNA sintético	Micro pocillo	IgG	120 min	Cuantitativa	Curva	0–200 UI/mL	20 UI/mL
IFI	dsDNA nativo	CrithideaLucilliae	IgG	60 min	Cualitativa	Ninguna	N/A	1/10

Modificada de Infantino M y cols. [33]

Con respecto al sustrato, CLIA [18], [31] y MPIA [19] utilizan dsDNA sintético unido a partículas magnéticas o paramagnéticas. FEIA [17] y EIA [14], emplean dsDNA sintético o purificado unido a micro pocillos. En CLIFT, el dsDNA está expuesto en el quinetoplasto del hemoflagelado *crithidia luciliae* [15].

El conjugado, en general, permite la detección de anticuerpos de clase IgG; si bien hay excepciones, por ejemplo, algunos equipos de EIA detectan anti-dsDNA de clase IgG e IgM [Kallestad^TM^] [32] y otros, permiten la detección de anti-dsDNA de clase IgA, IgG e IgM [Orgentec] [33].

La Tabla 1 muestra otras características de los métodos de análisis más frecuentes en los laboratorios clínicos europeos, de acuerdo a UK-NEQAS, información suministrada por los proveedores los reactivos e Infantino y cols. [33]. A continuación, se comentan algunas de las características:

los tiempos de análisis son más largos en las técnicas de EIA y FEIA que en las de MPIA y CLIA. Cuando CLIFT, se realiza manualmente, tarda aproximadamente 60 minutos; si bien, este tiempo es variable dependiendo de la automatización y la lectura al microscopio de fluorescencia.
los métodos FEIA, MPIA y CLIA permiten una carga continua de muestras; mientras que EIA y CLIFT trabajan en lotes.
las curvas de calibrado son estables durante un periodo de tiempo variable en FEIA, MPIA y CLIA; mientras que en EIA debe realizarse una curva por lote de muestras y en CLIFT no existe curva de calibrado [en cada lote se realiza un control negativo y positivo].
la detección es cuantitativa en todos los métodos excepto en CLITF, que es semicuantitativa con diluciones seriadas de las muestras. Aunque, en cualquiera de los métodos empleados, la expresión de resultados puede reducirse a cualitativa [positivo/negativo] utilizando un punto de corte establecido por el propio laboratorio o por el proveedor.
– el rango analítico varía entre los diferentes proveedores; siendo más amplio, en general, en los métodos de CLIA.

Por otra parte, los puntos de corte y valores de referencia deben ser establecidos en cada laboratorio. La tabla recoge valores de referencia provistos por el proveedor. Como puede observarse, a pesar de que todos los métodos se referencian a un estándar internacional, persiste variabilidad en puntos de corte y rangos de referencia; lo que demuestra defectos de estandarización.

El estudio comparativo de diferentes equipos entre los métodos recogidos en la tabla, ha demostrado un grado de acuerdo de moderado a sustancial entre ellos, con índices kappa comprendidos entre 0,47 y 0,68; sensibilidades clínicas para LES variables entre 5,7% [CLIFT] y 33,3% [EIA]; especificidades entre 89,8% [MPIA] y 98,8% [CLIFT]; razón de verosimilitud positiva entre 2,93 [MPIA] y 17,6 [CLIFT] y una razón negativa de verosimilitud poco significativa para cualquiera de los métodos que osciló entre 0,71 [EIA] y 0,96 [33].

Como era de esperar y de acuerdo a la experiencia existente, CLIFT es menos sensible, pero posee mayor especificidad y cuando es positiva, confiere al paciente la mayor probabilidad de poseer LES [razón de verosimilitud positiva más elevada] [34], [35].

Por ello, el Ministerio de Sanidad y Consumo Español recomienda la determinación de anticuerpos anti-dsDNA mediante CLIFT a dilución 1:10 en pacientes con anticuerpos antinucleares para el diagnóstico del LES [36]. Es posible, utilizar CFLIT como técnica de segunda línea, método confirmatorio, después de obtener un resultado positivo por una técnica cuantitativa automatizada de inmunoanálisis. En este caso, se recomienda informar ambos resultados, aunque exista discrepancia entre ellos [37]. En general, no se considera adecuada la técnica de inmunotransferencia “*blotting”* para el análisis de anti-dsDNA [22].

Además, las guías reflejan que el informe debe incluir el método utilizado y los valores de referencia del laboratorio para los controles sanos y pacientes con LES [3], [37].

Consideran que en personas con clínica de LES, una prueba de anticuerpos antinucleares positiva y un título elevado de anti-dsDNA, el LES es la primera opción diagnóstica [36]. Una vez establecido el diagnóstico y para la monitorización de la actividad lúpica pueden emplearse técnicas cuantitativas; informándose los resultados cuantitativos y empleándose el mismo método durante el seguimiento, siempre que sea posible [37].

En nefritis lúpica activa, un estudio reciente compara métodos de Farr, ELISA, FEIA y CLIA; demostrando que Farr y FEIA poseen sensibilidades comparables del 95%. Respecto a la actividad lúpica, Farr y FEIA se han comportado de forma similar [38].

#### Estandarización

El primer estándar internacional para anti-dsDNA, Wo/80 fue elaborado por la Organización Mundial de la Salud (WHO) en 1985 [39]. Este reactivo de referencia asignó Unidades Internacionales y mejoró la comparabilidad entre los test y los laboratorios; pero actualmente está agotado.

El Comité de expertos en estandarización y la WHO ha preparado y validado un nuevo reactivo de referencia, el denominado Reactivo de referencia anti-dsDNA (oligoespecífico) para lupus 15/174. Posee una potencia nominal de 100 unidades/ampolla; pero no es equivalente al primer estándar internacional (Wo/80) y no puede considerarse una continuidad del mismo. Está disponible en NIBSC (https://www.nibsc.org/ products/ brm_ product_ catalogue/detail_ page.aspx? catid=15/174) [40].

### Utilización clínica

#### Diagnóstico

Los anticuerpos anti-dsDNA forman parte de los criterios de clasificación de LES, como puede observarse en la Tabla 2. En ella se presenta la definición y el peso relativo de los anti-dsDNA en cada uno de los criterios existentes [criterios ACR 1982, criterios ACR revisados 1997, criterios SLIIC 2012 y ACR-EULAR 2019 [41], [42], [43], [44].

**Tabla 2:** Consideración de los anticuerpos anti-dsDNA en diferentes criterios de Lupus Eritematoso Sistémico.

Criterio	Especificaciones	Peso relativo
ACR 1982^a^	Título anormal de anti-ADN nativo	1/11 criterios
ACR 1997^a^	Título anormal de anti-ADN nativo	1/11 criterios
SLICC 2012^b^	Anti-dsDNA por encima del rango normal del laboratorio, excepto ELISA: 2 veces por encima del rango normal de referencia.	1/17 criterios
ACR-EULAR 2018^c^	Anti-dsDNA de especificidad elevada	1/22 criterios

^a^Clasificación LES cuando: el paciente posee cuatro o más de un total de 11 criterios, seriados o simultáneos, durante un intervalo indefinido de observación [41], [42]. ^b^Clasificación LES cuando: el paciente satisface cuatro de los 17 criterios [11 clínicos y 6 inmunológicos] incluyendo al menos 1 criterio clínico y 1 inmunológico, de forma seriada o simultánea; o cuando el paciente tiene una nefritis confirmada por biopsia compatible con LES y anticuerpos antinucleares o anti-dsDNA positivos [43]. ^c^Clasificación de LES cuando: los anticuerpos antinucleares sean positivos a título >1/80, la puntuación total sea >10, se puntúe al menos un dominio clínico y dentro de cada dominio, se valore exclusivamente el criterio de mayor valor [Peso de anti-dsDNA: 6 puntos] [44].

En los criterios SLICC 2012, los anti-dsDNA se analizaron mediante radioinmunoanálisis, CLIFT y ELISA. El criterio anti-dsDNA se asoció al diagnóstico de LES con una sensibilidad del 57,1% y una especificidad del 95,9%, sobre una muestra de 716 pacientes con diferentes enfermedades autoinmunes o inflamatorias [43]. En LES juvenil [en relación a artritis idiopática juvenil] la sensibilidad fue del 52,2% y la especificidad del 100% [45].

En los criterios ACR-EULAR 2019 [Tabla 3], 22 criterios se agrupan en diferentes dominios; aportándose un peso variable a cada criterio. Un paciente será clasificado de LES, cuando alcance 10 puntos y tenga, además, anticuerpos antinucleares positivos a título igual o superior a 1/80 en células HEp-2 o test equivalente. Los criterios y dominios se presentan en la tabla1 y no están limitados en el tiempo, pueden haber ocurrido previamente en el curso de la enfermedad [histórico de anticuerpos antinucleares positivo]. Los criterios no deben justificarse por patologías diferentes al LES y debe puntuarse al menos un dominio clínico. Cuando hay más de un criterio en un dominio clínico o inmunológico, solo se valorará el que tenga más peso. Los anti-dsDNA se integran en el dominio de anticuerpos altamente específicos. En su selección se tuvieron en cuenta las sensibilidades y especificidades de los criterios SLICC 2012 [referidos en el párrafo anterior] y ACR 1982 [67% y 92%, respectivamente] [41], [46].

**Tabla 3:** Criterios ACR/EULAR 2019 de Lupus Eritematoso Sistémico.

Dominios clínicos	Puntos
**Dominio constitucional**	
Fiebre	2
**Dominio cutáneo**	
Alopecia no cicatricial	2
Úlceras orales	2
Lupus cutáneo subagudo o discoide	4
Lupus cutáneo agudo	6
**Dominio articular**	
Sinovitis o dolor en al menos dos articulaciones y >30 minutos de rigidez articular	6
**Dominio neurológico**	
Delirio	2
Psicosis	3
Convulsiones	5
**Dominio de serositis**	
Derrame pleural o pericárdico	5
Pericarditis aguda	6
**Dominio hematológico**	
Leucopenia	3
Trombocitopenia	4
Hemólisis autoinmune	4
**Dominio renal**	
Proteinuria >0.5g/24 horas	4
Nefritis lúpica de clase II ó V	8
Nefritis lúpica de clase III ó IV	10

Dominios inmunológicos	Puntos
**Dominio de anticuerpos antifosfolípidos**	
aCL o aβ2GP1 o anticoagulante lúpico	2
**Dominio de proteínas del complemento**	
C3 o C4 bajas	3
C3 y C4 bajas	4
**Dominio de anticuerpos altamente específicos**	
Anti-dsDNA	6
Anti-Sm	6

aCL, anti-cardiolipina; aβ2GP1, anti-β2 glicoproteína1; anti-dsDNA, anti-DNA de doble cadena; anti-Sm, anti-Smith [41].

#### Asociación

Numerosos trabajos han demostrado que los anti-dsDNA, son patogénicos en la nefritis lúpica [NL] y se asocian significativamente a ella [47]. Los anti-dsDNA ejercen su papel patogénico fijándose a lo largo de la membrana basal y matriz mesangial en forma de complejos inmunes circulantes formados por dsDNA y proteínas de la cromatina [48]; pero también, el anti-dsDNA circulante puede unirse directamente a estructuras renales (fragmentos de cromatina expuestos en la membrana basal o antígenos glomerulares con los que reacciona de forma cruzada). Estos dos mecanismos patogénicos pueden coexistir, e incluso predominar, uno u otro en las distintas fases de la nefritis lúpica [49], [50].

La asociación clínica con la nefritis lúpica se incrementa cuando los anti-dsDNA coexisten con anti-nucleosomas y anti-histonas; contribuyendo a diferenciarLES con NL de aquel sin NL y confiriendo severidad a la NL [51]. Otros anticuerpos que pueden asociarse a anti-dsDNA y NL son los anti-fracción-C1q del complemento y los anti-Sm [39]. Se ha descrito que la positividad de estos tres anticuerpos con concentraciones bajas de C3/C4 y una relación disminuida albúmina/globulinas sugiere y parece predecir, afectación renal [52], [53]. Los más recientes, anticuerpos anti- proteínas de alta morbilidad que se unen al dsDNA parecen correlacionarse, igualmente, con anti-dsDNA y nefritis lúpica [53].

Algunas guías proponen monitorizar lar respuesta al tratamiento de la NL con niveles de anticuerpos anti-dsDNA y complemento, análisis del sedimento urinario, proteinuria en orina de 24 horas o cociente proteína/creatinina y creatinina sérica [54]. Sin embargo, otras han considerado que la especificidad de los anti-dsDNA con respecto a la NL es baja y poseen una utilidad limitada para el seguimiento de la misma [36].

#### Seguimiento

Las concentraciones de anti-dsDNA y componentes C3 y C4 [C3/C4] del complemento, actúan como marcadores serológicos de actividad en el LES. Así, y para la evaluación de la actividad, es adecuado evaluar conjuntamente el título de anticuerpos anti-dsDNA y los niveles de C3/C4 [55]. Si bien, hay que considerar, que la determinación de anticuerpos anti-dsDNA no va a predecir ni diagnosticar por sí solo un brote de actividad [3]. Los intervalos de medida se ajustarán a la situación clínica y serán, por tanto, variables. Si la enfermedad está en remisión clínica y analítica, se sugiere un seguimiento cada 6–12 meses, dependiendo del tiempo de evolución de la enfermedad y la intensidad del tratamiento. En pacientes clínicamente quiescentes, pero con criterios analíticos de actividad mantenidos, se sugiere un seguimiento más estrecho, cada 3–4 meses [3], [36], [55].

Los niveles de C3/C4 se modifican por el propio embarazo; no obstante, y a pesar de ello, se considera qué durante el embarazo, la determinación de C3/C4 y anti-dsDNA permite monitorizar la actividad lúpica; debiendo realizarse su análisis ante sospecha de un brote de actividad [36]. Andreoli y cols, indican que la elevación de anti-dsDNA y la disminución de C3/C4 confieren a la gestante un riesgo aumentado de brote de LES [OR 5.3], al igual que incrementa el riesgo de pérdida fetal [56].

Los anticuerpos anti-dsDNA y C3/C4 están incluidos en índices clínicos de actividad del LES. En la Tabla 4 se presenta uno de los más utilizados, SLEDAI-2K descrito por Gladman [57] que recoge la presencia o ausencia de cada uno de los descriptores de actividad en los 30 días precedentes. SRI-50, basado en SLEDAI-2K, definemejoría clínica importante sobre el nivel basal al año, cuando los descriptores de SLEDAI-2K disminuyen > 50% [58]. Existen otros índices de actividad como BILAG [*British Isles Lupus AssessmentGroupIndex*] y BILAG 2004, ECLAM [*EuropeanConsensus Lupus ActivityMeasurement*], SLAM [*Systemic Lupus ActivityIndex*]ySLAQ [*SystemicLupus ActivityQuestionnaire*] que no siempre van a considerar la elevación de anti-dsDNA y la hipocomplementemiaentre sus descriptores [59]. En 2016 un comité de expertos ha considerado, por consenso, la presencia de anti-dsDNA, junto con hipocomplementemia, como marcadores de actividad del LES (definidos como por encima o por debajo del valor de referencia del laboratorio). El 93% de los expertos han recomendado añadir estos marcadores a la definición de remisión de LES [60].

**Tabla 4:** Índice de actividad de la enfermedad Lupus Eritematoso Sistémico [SLEDAI 2000] o SLEDAI-2K.

Descriptor	Puntuación
Convulsiones	8
Psicosis	8
Síndrome orgánico cerebral	8
Alteración visual	8
Alteración nervios craneales	8
Cefalea lúpica	8
Accidente vascular cerebral	8
Vasculitis	8
Artritis	4
Miositis	4
Clindros urinarios^a^	4
Hematuria^b^	4
Proteinuria^c^	4
Piuria^d^	4
Rash	2
Alopecia	2
Úlceras mucosa	2
Pleuritis	2
Pericarditis	2
Complemento bajo^e^	2
Anti-dsDNA elevado^f^	2
Fiebre	1
Leucopenia^g^	1
Tromcobitopenia^h^	1

^a^Cilindros granulares o hemáticos; ^b^más de 5 hematíes/campo; ^c^más de 0,5g proteínas/24 horas; ^d^más de leucocitos/campo; ^e^complemento CH50, C3 o C4; ^f^anti-dsDNA superior al rango de referencia; ^g^leucocitos < 3 × 10^9^/L; ^h^plaquetas <100 × 10^9^/L. Dependiendo del descriptor se considera reciente comienzo o persistencia.

Actualmente, se está intentando diferenciar actividad reducida y remisión del LES. Se tiende a considerar como remisión completa cuando el LES está inactivo sin medicación alguna o solo con anti-maláricos, como la hidroxicloroquina. Para actividad reducida existen diversas definiciones que tienen en cuenta SLEDAI-2K y los fármacos necesarios para mantener la actividad reducida. Se han definido, actividadmínima [MDA], actividad baja [LDA] y estado de actividad baja del LES [LLDAS]. La actividad serológica [medida con anti-dsDNA y C3/C4] está incluida exclusivamente en LLDAS. Mientras que MDA y LDA, solo tienen en cuenta variables clínicas [61].

En una guía reciente del Colegio Mexicano de Reumatología, se recomienda tomar en consideración los niveles bajos de complemento, la elevación de las concentraciones de anti-dsDNA y la elevación discreta de proteína C reactiva, para determinar en el paciente con LES, si la presencia de fiebre se asocia o no a la actividad de la enfermedad [54].

Para analizar la respuesta al tratamiento pueden emplearse distintos índices de respuesta [58]. No obstante, el papel de los anti-dsDNA en la monitorización del tratamiento es controvertido. Las experiencias son muy variables dependiendo de diversos factores, entre ellos los equipos de medida. Algunos expertos opinan que con una estandarizaran adecuada, bien solos o formando parte de un índice compuesto "score" con parámetros clínicos o analíticos, podrían monitorizar la respuesta al tratamiento (biológico o no-biológico) [1], [62], [63].

### Orientaciones de uso

En este último apartado, los autores proponen orientaciones para una utilización clínica adecuada de los anti-dsDNA, derivadas de la información analizada.

El interés clínico y patogénico de los anti-dsDNA se incrementa cuando se detectan anti-dsDNA de afinidad y avidez elevadas. En general, se considera que se detectan anticuerpos alta avidez y afinidad con las técnicas de Farr [hoy, en desuso] y CLIFT, lo que les confiere una mayor especificidad. Por ello, se recomienda utilizar CLIFT en el diagnóstico de LES; si bien, puede utilizarse como técnica de segunda línea después de una técnica cuantitativa positiva. En caso de no poder realizar CLIFT, se considerarán resultados positivos los superiores a dos veces el límite superior del rango de referencia del grupo control. El clínico debe conocer el método utilizado y los rangos de referencia del laboratorio.

Para el diagnóstico y ante la sospecha clínica de un posible LES, los anti-dsDNA deben determinarse en pacientes con anticuerpos antinucleares positivos; informándose el resultado de ambos anticuerpos. Excepcionalmente, y ante una sospecha clínica elevada, podrían determinarse anti-dsDNA directamente; ya que pueden detectarse anti-dsDNA en pacientes con anticuerpos antinucleares negativos, en función de la técnica y el punto de corte utilizados; ya que influyen los métodos de fijación y las características de los métodos analíticos. Así, por ejemplo, en las células HEp-2, el dsDNA se encuentra en el núcleo unido a histonas y otras proteínas nucleares y se fija al soporte de vidrio con solventes orgánicos; mientras que en los métodos específicos, anti-dsDNA nativo, purificado o recombinante se une de forma aislada a un soporte sólido, generalmente plástico.

Los anti-dsDNA solo poseen utilidad diagnóstica en el LES y siempre deben interpretarse en el contexto clínico. Forman parte de los criterios de clasificación inmunológicos por su especificidad elevada. Una concentración elevada de anti-dsDNA en personas con clínica de LES y prueba de anticuerpos antinucleares positiva, sugiere LES como primera opción diagnostica; sin embargo, un resultado negativo no va a excluir LES, dada su pobre sensibilidad y razón de verosimilitud negativa.

Se utilizará la determinación de anti-dsDNA cuantitivo para el seguimiento del paciente con LES, recomendándose emplear el mismo método en todo el seguimiento.

Los anti-dsDNA se asocian a actividad lúpica, especialmente cuando se detectan a concentraciones elevadas, con métodos de especificidad elevada y se asocian a algún otro signo clínico o de laboratorio de actividad en el paciente. Una elevación de anti-dsDNA no predice ni diagnostica por sí sola una activación de la enfermedad.

Los anti-dsDNA y los niveles de C3/C4 del complemento están incluidos en índices de actividad de LES y se recomienda su evaluación conjunta ante la sospecha de un brote de actividad, incluidas gestantes con LES.

Los anti-dsDNA se correlacionan con NL y poseen una utilidad relativa para su diagnóstico y actividad. Al igual que con la actividad, concentraciones y especificidades elevadas, mejoran la asociación y la utilidad clínica de los anti-dsDNA en NL; pero deben interpretarse en conjunción con otras medidas de enfermedad renal.

## Conclusiones

Existe una amplia variabilidad en los métodos analíticos empleados clínicamente para el análisis de anti-dsDNA; así como déficits en su estandarización. No todos los métodos van a detectar anticuerpos con el mismo grado de avidez y afinidad. Los anti-dsDNA de avidez y afinidad elevadas en pacientes con ANA positivos, poseen una elevada especificidad diagnóstica para LES. En el seguimiento, es interesante la cuantificación de estos anticuerpos, preferiblemente con el mismo método analítico (dado los déficits de estandarización). Destacar, por último, que la variabilidad y los avances en los métodos de medida van modificando sus características clínicas.
